# Sleep quality and sleep deprivation: relationship with academic performance in university students during examination period

**DOI:** 10.1007/s41105-023-00457-1

**Published:** 2023-04-10

**Authors:** Maria Suardiaz-Muro, Manuel Ortega-Moreno, Miguel Morante-Ruiz, Manuel Monroy, Miguel A. Ruiz, Pilar Martín-Plasencia, Antonio Vela-Bueno

**Affiliations:** 1 Department of Biological and Health Psychology, School of Psychology, Universidad Autónoma de Madrid (UAM), Madrid, Spain; 2 Psychiatry Service, Hospital Universitario de Móstoles, Madrid, Spain; 3 Internal Medicine Service, Hospital Universitario Fundación Jiménez Díaz, Madrid, Spain; 4 Department of Social Psychology and Methodology, School of Psychology, Universidad Autónoma de Madrid, Madrid, Spain; 5 Department of Biological and Health Psychology. School of Psychology, Universidad Autónoma de Madrid, Madrid, Spain; 6 Department of Psychiatry, Pennsylvania State University, Hershey, USA; 7 Department of Neuropsychology. Rehabilitation Service, Fundación Instituto San José, Madrid, Spain

**Keywords:** Academic performance, Daytime sleepiness, Napping, Sleep deprivation, Sleep quality, University students

## Abstract

The beginning of the university brings together maturational, psychosocial and academic changes that make university students more prone to suffer from insufficient or poor quality sleep, which can negatively influence their academic performance. The period of taking exams is a key part of the academic year. However, there are few studies that analyze sleep during this period of time. Our aim is to study the association of sleep quality and sleep deprivation with academic performance during the examination period. A descriptive, cross-sectional and correlational study was carried out with the participation of 640 subjects in the first three years of five faculties belonging to the Universidad Autónoma de Madrid. The instrument used consisted of a questionnaire that included sociodemographic and academic data, Pittsburgh Sleep Quality Index, Epworth Sleepiness Scale and information about the academic performance. During the examination period, a positive association was found between sleep quality and academic performance. University students slept less than desired, both on weekdays and weekends, and the sleep debt during the week was associated with a worse students’ perception of their academic performance. In total, 61.3% of the students believed that their performance would improve by getting more sleep. In addition, low drowsiness and napping were also found. In conclusion, during periods of greater academic demand, an insufficient sleep and poor quality is commonly observed, affecting negatively to their academic performance. Actually, about 2/3 of our subjects believed that their performance would improve by getting more sleep.

## Introduction

The beginning of the university years is associated with an increased risk of having problems with regularity, duration, and quality of sleep, which in many cases result in an insufficient sleep. The most obvious consequence is excessive daytime sleepiness [1, 2]. Taking into account the essential role of sleep in the general homeostasis, such problems may result, among others, in a poor daytime functioning and negatively impact over the academic performance (AP) [3, 4].

Two types of factors contribute to the above mentioned sleep problems: general and specific. Among the general factors there are those of a biological nature, associated with the maturational changes of the life cycle [5], and those of a psychosocial nature, such as the ones linked to the processes of individuation and socialization [2]. On the other hand, specific factors include academic demands [5].

In the last decades, there has been increasing interest in the relationship between sleep and AP in university students. Generally speaking, it appears to be a relationship between inadequate, insufficient or irregular sleep and AP [3, 4, 6]. The majority of studies refer to the entire academic year while a few take into account the period prior to exams or exam days, which involves stressful situations [7–12].

The purpose of this paper is to understand the effect of quantity and quality of sleep of university students during the period of preparation for and taking final exams and to determine its influence on students' perception of their AP.

### Objectives and hypotheses

Objectives:

- To study the relationship between sleep quality, as measured by the Pittsburgh Sleep Quality Index (PSQI), and AP.
- To identify the PSQI components that are related to AP.
- To study the influence of sleep deprivation on AP.

Hypothesis:

- Sleep quality is positively related to AP.
- Being a good sleeper (PSQI ≤ 5) is associated with better AP.
- PSQI components contribute differentially to AP.
- Partial sleep deprivation is negatively related to good AP.

## Materials and methods

Descriptive, cross-sectional and correlational study. Subjects were randomly selected, by invitation to participate in the study.

Inclusion criteria:

- Students of the Autónoma University of Madrid (UAM) (Public University ranked fourth in the 2023 ranking of Spanish universities).
- They belonged to five faculties representative of different fields of study: Sciences (Mathematics and Physics), Medicine, Psychology, Law and Philosophy.
- They were in the first three academic years, which are those that require the greatest effort to adapt to the new environment.
- Subjects from 18 to 26 years of age, age typical of university studies.

Exclusion criteria:

- Subjects who were working at the same time as studying.

The instrument consisted of a computerized survey form developed by the authors using Google Drive^®^ software, which included validated scales and questionnaires. It was sent to the institutional mail of the target subjects during the period of the final exams of the course (May and June). All subjects gave informed consent.

The study was approved by the UAM research ethics committee. The information was collected guaranteeing confidentiality and anonymity in accordance with current national law (LOPD15/1999).

The following variables were measured:

- Sociodemographic: age and sex.
- Anthropometric: weight and height.
- Academic: faculty, course and shift class schedule.
- Sleep habits: bedtime, waking time and rising time, sleep latency and napping were collected. In addition, subjects reported the hours they thought they had slept each night (item 4 of the PSQI) and the hours of sleep they would like to sleep.
- Sleep debt: this concept has been defined and used by several authors as the discrepancy between self-reported ideal sleep time and real sleep time [13, 14]. Therefore, from the sleep habits subset of variables previously indicated, we calculated sleep debt as the difference between the hours of sleep that subjects reported sleeping each night (item 4 of the PSQI) and their reported desired sleep.
- Sleep quality: the Pittsburgh Sleep Quality Index [15, 16] was used. It consists of 19 self-administered items that provide a total score on sleep quality during the last month, ranging from 0 to 21. Lower scores indicate better sleep quality. The cut-off point for being a good sleeper is a score of less or equal five. It also provides information on seven components: subjective sleep quality, sleep latency, sleep duration, habitual sleep efficiency, sleep disturbances, hypnotic medication usage, and daytime dysfunction.
- Daytime sleepiness: the most widely used scale for measuring daytime sleepiness, the Epwoth Sleepiness Scale (ESS) [17, 18], was used. It consists of 8 self-applied items that assess the likelihood of falling asleep in different situations. The overall score ranges from 0 to 24. The higher the score, the greater the degree of daytime sleepiness, with excessive daytime sleepiness defined at an overall score bigger than ten.
- Stimulant beverages intake: it was assessed by asking the subjects how often they drank such beverages, according to the following categories: none, ocasionnally, daily, more than one a day.
- Academic performance: consists of two questions with a Likert-type response format in which information is collected on the student's self-perception of: (a) his/her AP (How do you think your AP is: very bad, bad, fair, normal, good and very good), (b) the link between this and his/her sleep (Do you think your AP would improve if you slept more? YES/NO/sleep enough).

We assessed AP as judged by the students themselves. This was decided based on several considerations:

- Different teachers use different strategies for measuring it.
- Differences among academic topics (eg.: mathematical vs. verbal contents).
- Differences among schools (eg: literary vs technical). It is known that each faculty has developed over time their own way of assessing performance and it is difficult to compare values obtained from different schools. This is even more evident in countries like Spain where assessment is made based on typical performance (with a cut-point decided by the teacher) and there is no tradition in using normative assessment (based on the distribution of class-room scores).
- Also, the workload and academic demands vary among different type of schools and this has an impact on the differences regarding sleeping patterns and habits.

This approach based on self assessment has been adopted nowadays in clinical studies [19].

### Statistical analyses

In order to homogenize the sample and reduce possible selection biases, criteria for inclusion and exclusion of subjects were determined. For reasons of size of some subgroups, the analyses were performed by grouping the categories of the dependent variable (AP) into "poor", "normal" and "good". The negative categories (very poor, poor and fair) were grouped as "poor AP" and the positive categories (good and very good) as "good AP".

Frequencies and percentages were used to describe categorical variables, and Pearson's chi-square and adjusted standardized residuals were used for comparisons. Means and standard deviations were used to describe quantitative variables (unless otherwise specified) and comparisons were made using t-tests, in the case of two groups, or fixed-effect ANOVA with multiple comparisons, adjusted by the Bonferroni procedure. Statistically significant differences were associated to an alpha level of 5% (*p* < 0.05). IBM SPSS Statistics version 26 software was used to perform the analyses.

## Results

### Sample description

A total of 640 subjects, ranging in age from 18 to 23 years (19.78 ± 1.25) completed the survey. Table 1 shows the characteristics of the subjects.

**Table 1:** Sample description

	Statistic	
SOCIODEMOGRAPHIC		
Age	$\overline{\mathrm{X }}\pm \mathrm{SD }$	19.78 ± 1.25
Sex		
* Male*	*n* (%)	170 (26.6)
* Female*	*n* (%)	470 (73.4)
ANTHROPOMETRIC		
BMI	$\overline{\mathrm{X }}\pm \mathrm{SD }$	21.59 ± 3.05
* Under weight*	*n* (%)	81(12.7)
* Normal weight*	*n* (%)	485 (75.8)
* Over weight*	*n* (%)	59 (9.2)
* Obesity*	*n* (%)	15 (2.3)
ACADEMIC		
School		
* Sciences (Mathematics/Physics)*	*n* (%)	150 (23.4)
* Medicine*	*n *(%)	181 (28.3)
* Psychology*	*n* (%)	80 (12.5)
* Law*	*n* (%)	73 (11.4)
* Phylosophy*	*n* (%)	156 (24.4)
Course		
* 1st*	*n* (%)	213 (33.3)
* 2nd*	*n* (%)	235 (36.7)
* 3rd*	*n* (%)	192 (30.0)
Shift class schedule		
* Morning*	*n* (%)	464 (72.5)
* Afternoon*	*n* (%)	176 (27.5)
SLEEP QUALITY		
* Global Score (PSQI)*	$\overline{\mathrm{X }}\pm \mathrm{SD }$	6.98 ± 3.41
* Good sleepers (PSQI ≤ 5)*	*n* (%)	244 (38.1)
* Poor sleepers (PSQI > 5)*	*n* (%)	396 (61.9)
SLEEP DEPRIVATION		
Sleep debt		
* Weekdays*	*n* (%)	2:09 ± 1.18
* Weekend*	*n* (%)	1:11 ± 1.29
Daytime sleepiness		
* Global Score ESS*	$\overline{\mathrm{X }}\pm \mathrm{SD }$	7.07 ± 4.01
* Normal (ESS ≤ 10)*	*n* (%)	528 (82.5)
* Excessive (ESS > 10)*	*n* (%)	112 (17.5)
Do you think your AP would improve if you slept more?		
* Yes*	*n* (%)	392 (61.3)
* No*	*n* (%)	83 (13.0)
* I sleep enough*	*n* (%)	165 (25.8)
Nap		
* Yes*	*n* (%)	229 (35.8)
* No*	*n* (%)	411 (64.2)
STIMULANT BEVERAGES INTAKE		
* None*	*n* (%)	237 (37)
* Occasionally*	*n* (%)	80 (12.6)
* Daily*	*n* (%)	127 (19.8)
* More than one a day*	*n* (%)	196 (30.6)

Mean time values in h:min format, standard deviations as hours and decimals

$\overline{\mathrm{ X } }:$ Mean, *SD* standard deviation

### Relationship between sleep variables and academic performance

Sleep quality showed significant differences between the three AP groups (*F* = 17.804, *p* < 0.001; see Table 2). Higher overall scores on the PSQI, indicating worse sleep quality, were associated with worse AP (and vice versa). The categorization of subjects into good/poor sleepers also showed significant association with AP (*χ*^2^ = 0.191, *p* < 0.001). Among the good sleepers, a higher percentage of students with good AP was found than in the two other groups in both separately or added together.

**Table 2:** Sleep quality and differences on academic performance

	AP						Statistic	*p*
	Poor AP		Normal AP		Good AP			
	$\overline{X }$	SD	$\overline{X }$	SD	$\overline{X }$	SD		
Sleep quality (global score PSQI)	8.23*	3.52	7.26*	3.40	6.28*	3.20	17.804	< 0.001

	*n*	%	*n*	%	*n*	%		
Good sleepers PSQI ≤ 5	35	14.3	58	23.8	151	61.9	0.191	< 0.001
Poor sleepers PSQI > 5	107	27.0	118	29.8	171	43.3		

*AP* Academic performance, $\overline{\mathrm{X } }:$ Mean, *SD* standard deviation, *p*: significance level

*When the group differs from all other

The sleep quality components showed significant differences with respect to the AP groups, particularly the components of: subjective sleep quality (*F* = 19.03, *p* < 0.001), sleep duration (*F* = 8.54, *p* < 0.001), sleep efficiency (*F* = 10.64, *p* < 0.001), sleep disturbances (*F* = 3.84, *p* = 0.022) and daytime dysfunction (*F* = 16.41, *p* < 0.001). Sleep latency (*F* = 1.41, *p* = 0.114) and hypnotic medication use (*F* = 1.08, *p* = 0.34) showed no association.

Sleep deprivation variables showed that weekday sleep debt was associated with a worse AP (*F* = 3.77, *p* = 0.024; see Table 3). We observed significant differences between the AP groups and students' estimation of the influence of sleep on their AP (*χ*^2^ = 16.9, *p* < 0.001). In all the three performance groups, a higher percentage of subjects believed that their performance accomplishment would improve by getting more sleep than the percentage of those who did not believe so or those who felt they got enough sleep. In total, 61.3% responded affirmatively to the question about the influence of sleep on their AP, 15.6% belonged to the group with a poor perception of AP, and 18.8% and 26.9% to the normal and to the good perception group, respectively.

**Table 3:** Sleep deprivation and differences on academic performance

	Poor AP		Normal AP		Good AP		Statistic	*p*
	Mean	SD	Mean	SD	Mean	SD		
SLEEP DEBT								
* Weekdays*	2:40^B^	1.31	2:19^AC^	1.33	2:04^B^	1.28	3.77	0.024
* Weekend*	1:22	1.29	1:10	1.25	1:06	1.31	1.63	0.196
DAYTIME SLEEPINESS (global score ESS)	7.13	4.10	7.40	4.08	6.87	3.94	1.02	0.360

	*n*	%	*n*	%	*n*	%		
* Normal (ESS ≤ 10)*	114	80.3	147	83.5	267	82.9	0.65	0.722
* Excessive (ESS > 10)*	28	19.7	29	16.5	55	17.1		
STUDENTS WHO ANSWERED “YES” TO THE QUESTION “DO YOU THINK YOUR AP WOULD IMPROVE IF YOU SLEPT MORE?”	100^B^	70.4	120^A^	68.2	172	53.4	16.9	< 0.001
NAP								
* Yes*	44	31.0	61	34.7	124	38.5	2.56	0.278
* No*	98	69.0	115	65.3	198	61.5		

*AP* Academic performance, $\overline{\mathrm{X } }:$ Mean, *SD* standard deviation, *p* significance level

A, B, C: groups with no significant difference (*p* > 0.05) are labelled with the letter corresponding to the non-differing groups (from left to right)

## Discussion

Our results, like those of other authors [7, 12], indicate that during the exam period of time, the percentage of students who sleep poorly is higher than those who sleep well. Most studies show that better sleep quality is associated with a better AP [20–25]. We found that, during final exams, there is a positive association between both variables, indicating that as sleep quality improves, the student's perception of their AP improves as well. These results are consistent with those of other authors [7, 10, 11]. Ahrberg et al. [7], studied this association in three different moments of the course: during the semester, the pre-exam period and the post-exam period, finding an association between AP and sleep quality only in the pre-exam period.

Five components of the PSQI showed significant association between sleep quality and AP: subjective sleep quality, sleep duration, sleep efficiency, sleep disturbances and daytime dysfunction. Other studies also found this association [20, 22, 24, 26, 27]. Our study has not demonstrated association with AP in the components of sleep latency and hypnotic medication use. In the literature it is shown that the data regarding both components are controversial [24, 26].

Our results suggest that students sleep on average less than desired, both on weekdays and on weekends. The literature collects few studies that include student self-perception of getting sufficient sleep [28–30].

Increased sleep debt on weekdays worsens perception on AP. In contrast, on the weekend, we found no significant differences between the three performance groups. This can be explained by the fact that students during the exam period organize their schedules in a more demanding manner during the week. However, at the weekend they tend to recover the sleep debt accumulated during the week. Bahammam et al. [28] obtained results similar to the data presented in this work. In their study, the proportion of students who considered getting enough sleep was higher in students who had better AP. Perception of sufficient sleep was one of the significant predictors of AP.

The most obvious consequence of inadequate sleep in terms of duration, quality and regularity is daytime sleepiness [2]. In our work the presence of sleepiness (18%) is lower than in other similar studies, ranging from 28.7% to 52% [22, 25, 31–34]. We didn´t find any association between daytime sleepiness and stimulant beverages.

Most research that has studied the influence of daytime sleepiness on AP suggests a negative influence [22, 28, 31]. However, there are studies that have not demonstrated an association between the two variables [25, 32]. In the present work, sleepiness showed no significant association with AP. Students showed poor sleep quality and insufficient sleep, yet they did not present daytime manifestations of excessive sleepiness. This could be explained by the increased hyperarousal associated with the exam period, which could counteract and avoid the expression of sleepiness.

The most common way to cope with daytime sleepiness caused by insufficient nighttime sleep is napping. In most of the studies in the literature, the percentage of students who reported napping at least once a week at some time during the day was higher than those who reported never or sporadic napping [23, 25, 31, 33, 35, 36]. Only a few studies have shown that the percentage of students who reported napping was lower than those who did not [30, 37]. Only Alqarni et al. [38] included in their study a question about the tendency of students to nap during the final exam period, finding that the percentage who reported not napping was higher than those who did. In our study we found similar results. Thus, 64.3% of the students indicated that they did not nap. A role for the aforementioned hyperarousal could explain this finding.

There are few existing studies in the literature that include aspects such as student's knowledge or self-perception of the contribution of sleep in AP [21, 29, 35, 36, 38, 39]. These studies use different approaches. Gomes et al. [21] analyzed variables associated with a perception of greater impact of sleep on AP. Gikunda et al. [39] asked subjects to indicate their degree of agreement with statements about the impact of sleep on AP. Others have asked subjects about the number of hours they consider necessary for better AP [35, 36]. Some studies have analyzed whether subjects consider their performance on an exam to be influenced by sleep [29, 38]. Thus, in the study by Kazim & Abrar [29] 36.5% reported that their performance was affected by inadequate sleep. Alqarni et al. [38] included items asking students about what they thought their AP would be, depending on if they slept poorly and if they slept well. The percentage of students who answered that their performance would be poor or very poor was 45.8% if the first case and 2.3% in the second case. In our study we asked students to indicate what they thought their AP would be like if they slept more. About 61% considered that their AP was affected by sleep indicating that it would improve if they slept more.

We assume that our work has some limitations. On the one hand, the selection of the subjects was made by invitation to participate in the study, which may elicit the collaboration of subjects particularly interested in the subject. On the other hand, the use of self-report measures in data collection implies a certain subjectivity in the responses. Another limitation is that there could be mediating variables in the relationship between sleep and AP that have not been taken into account. We lack information about sleep dirsorders because this is not a clinical study so we can not exclude the posiblilty of some type of influence of those disorders on our results. Although no subject recognized to suffer any relevant pathology, we cannot rule out that they don’t suffer any condition interfering in our results. Other conditions involving hyperarousal, such as stress, depression and anxiety, could be influencing our results too. In addition, the study design does not allow causal relationships to be established. Finally, the findings obtained in the students of the first three years and five Faculties cannot be generalized to other populations.

## Conclusion

The results of our study show that in a period of greater academic demand, such as the one of final exams, the sleep of university students is insufficient and of poor quality, negatively affecting their AP. Actually, about 2/3 of our subjects believed that their performance would improve by getting more sleep.

Our results stress the importance of obtaining an adequate amount of good quality sleep to performe well. Education about this issue is crucial for making students, teachers and society in general aware of the contribution of sleep to the general homeostasis and health.
